# Repetitive speech elicits widespread deactivation in the human cortex: the “Mantra” effect?

**DOI:** 10.1002/brb3.346

**Published:** 2015-05-04

**Authors:** Aviva Berkovich-Ohana, Meytal Wilf, Roni Kahana, Amos Arieli, Rafael Malach

**Affiliations:** Department of Neurobiology, Weizmann Institute of Science234 Herzl St., Rehovot, 76100, Israel

**Keywords:** Default mode network, fMRI, global inhibition, mantra, meditation

## Abstract

**Background:**

Mantra (prolonged repetitive verbal utterance) is one of the most universal mental practices in human culture. However, the underlying neuronal mechanisms that may explain its powerful emotional and cognitive impact are unknown. In order to try to isolate the effect of silent repetitive speech, which is used in most commonly practiced Mantra meditative practices, on brain activity, we studied the neuronal correlates of simple repetitive speech in nonmeditators – that is, silent repetitive speech devoid of the wider context and spiritual orientations of commonly practiced meditation practices.

**Methods:**

We compared, using blood oxygenated level-dependent (BOLD) functional magnetic resonance imaging (fMRI), a simple task of covertly repeating a single word to resting state activity, in 23 subjects, none of which practiced meditation before.

**Results:**

We demonstrate that the repetitive speech was sufficient to induce a widespread reduction in BOLD signal compared to resting baseline. The reduction was centered mainly on the default mode network, associated with intrinsic, self-related processes. Importantly, contrary to most cognitive tasks, where cortical-reduced activation in one set of networks is typically complemented by positive BOLD activity of similar magnitude in other cortical networks, the repetitive speech practice resulted in unidirectional negative activity without significant concomitant positive BOLD. A subsequent behavioral study showed a significant reduction in intrinsic thought processes during the repetitive speech condition compared to rest.

**Conclusions:**

Our results are compatible with a global gating model that can exert a widespread induction of negative BOLD in the absence of a corresponding positive activation. The triggering of a global inhibition by the minimally demanding repetitive speech may account for the long-established psychological calming effect associated with commonly practiced Mantra-related meditative practices.

## Introduction

A ubiquitously practiced cognitive task, spanning thousands of years, and various spiritual traditions, is the simple act of repetitive silent speech, termed “Mantra” in eastern traditions. A Mantra (in Sanskrit, *manas* – mind, *tra* - tools or instruments, hence literally ‘an instrument of thought’) is a sound, word, or group of words whose repetition can exert calm, or mental quiescence, without the need for intense concentrative efforts (Feuerstein 2003). Mantra is being practiced within the wider context of meditation, which was generally conceptualized for neuroscience (Lutz et al. 2008) as a family of complex emotional and attentional regulatory strategies developed for various ends, including the cultivation of well-being and emotional balance. Mantra meditation has been linked experientially to a wide array of emotional and cognitive effects ranging from calm concentration and quiescence of the mental chatter, to deep absorption and mystical states (Goleman 1988; Travis et al. 2010).

An extensive body of data has accumulated using blood oxygenated level-dependent (BOLD) functional magnetic resonance imaging (fMRI) to study the effects of various meditation practices on brain activity, including silent Mantra meditation (reviewed by Cahn and Polich 2006; and more recently by Sperduti et al. 2012; and by Tomasino et al. 2012). Studies of Mantra meditation typically compare the Mantra meditation to control tasks, including pseudo-words and words silent repetition or generation. These control tasks are supposed to subtract language-related activations originating from the Mantra repetition without evoking an emotional response (Tomasino et al. 2012). In order to try to isolate the effect of silent repetitive speech, which is used in most commonly practiced Mantra meditative practices, on brain activity, we studied the neuronal correlates of simple repetitive speech in nonmeditators –that is, silent repetitive speech devoid of the wider context and spiritual orientations of commonly practiced meditation practices. We compared a simple task of covertly repeating a single word (the number “one”) to resting state activity, in 23 subjects, none of which practiced meditation before. Importantly, we did not choose as a meditation object a word that necessary had mantra-like qualities, as the word “one” has not been used in any meditation tradition, well pointed out by an anonymous reviewer. While mantras appear to be carefully selected for their physiological or spiritual qualities, we purposefully tried to isolate the effect of silent repetitive speech.

Here, we show that repetitive speech perse caused a unidirectional reduced activation in widespread cortical networks, including the default mode network (DMN) relative to resting state baseline. The DMN is a network that tends to reduce its activation during performance of externally oriented tasks (Raichle et al. 2001; Buckner et al. 2008; Preminger et al. 2011). It includes the medial prefrontal cortex, precuneus/posterior cingulate cortex, inferior parietal lobule, medial temporal lobe including the hippocampus, and lateral temporal cortex (Raichle et al. 2001; Buckner et al. 2008). The DMN is related in general to internally oriented thought processes, including evaluative self-reference (Gusnard et al. 2001), predicting and planning (Raichle and Snyder 2007; Preminger et al. 2011) and mind-wandering (Mason et al. 2007). Importantly, in many contemplative traditions, mind wandering is considered a distraction and a gateway to rumination, anxiety and depression (Sood and Jones 2013), and therefore its practice aims at reducing stimulus independent thought.

We hypothesized that part of the documented calming impact of Mantra mediation may be due to its repetitive speech aspect – resulting in a wide-spread cortical inhibition. We therefore examined here whether repetitive speech in nonmeditators may result in such BOLD reduction even when compared to resting state. Our results confirmed our hypothesis, by demonstrating that repetitive speech produced a global reduction in brain activity. This is in contrast to conventional cognitive tasks, where cortical reduced activation is complemented by positive BOLD activity (Mckiernan et al. 2003; Fox et al. 2005; Golland et al. 2007; Chang and Glover 2009). We further discuss the hypothesis that such global reduction was a result of nonlinear cortical gating mechanism, where minimal activation of a key “bottle-neck” region may inhibit wider competing processes (Marti et al. 2012).

## Materials and Methods

### Participants

Twenty three healthy participants (age 40 ± 9.5 years, eight female) underwent fMRI scanning; all were right handed by self-report and had no history of neurological disorders. Of these, twelve participants had breathing measurements taken during the MRI scan (described in section Respiration recording). All participants provided written informed consent for their participation. The experimental procedures were approved by the Ichilov hospital ethics committee. A separate group of 30 participants took part in a behavioral study (described in section Behavioral test).

### Stimuli and experimental design

**Figure 1:**
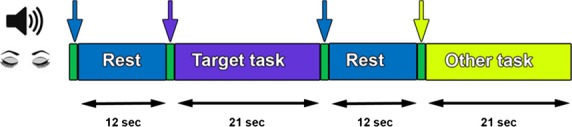
Experimental protocol. The figure depicts the time and sequence of conditions in each of the two experiments. A block design was used, and each condition was given five times in an experiment. Purple epochs represent the target task condition, which was Verbal Fluency (VF) in the first experiment, and Repetitive Speech (RS) in the second, in a counterbalanced manner. Each experiment consisted of additional experimental conditions, marked by yellow (two in the RS experiment, and three in the VF experiment). Participants performed the experiments with closed eyes and all epochs were indicated by brief (<1.1 sec) auditory cues (green epochs, marked by arrows).

The tasks reported here were parts of a larger study, all of it conducted in the same fMRI scan. We report here results from two fMRI experiments, both conducted with eyes closed: A. Repetitive Speech (“RS”) – silently repeating the word “one” (in Hebrew “Echad”) at a self-paced rate; and B. Verbal Fluency (“VF”) – silent generation of words that begin with a specific, randomly chosen, letter. Word generation was performed at a self-paced rate without vocalization. The VF and RS experiments had identical durations of activation blocks and rest. Thus, both conditions were in five blocks (21 sec each), which were interspersed with quiet rest (“Rest”) periods (12 sec each) (Fig.1).

The RS experiment lasted altogether for 8.25 min. There were five RS blocks (21 sec each) which alternated in pseudorandom order with two other conditions (all in five blocks of 21 sec each): a tone discrimination motor task and a thought-control condition. All the blocks were interspersed with Rest periods (12 sec each). The “VF” experiment lasted altogether 11 min. The five VF blocks (21 sec each) alternated in pseudorandom order with three other conditions (five blocks of 21 sec each block): an abstract thought task, and an imagery task, as well as a sentence-conjugation condition (which was used to independently define verbal regions, see section Regions of interest analyses).

In both experiments, the participant's head was placed on a foam cushion for stabilization, and MR compatible earphones (MR confon, Magdeburg, Germany) that substantially reduce external noise were placed on the ears. Participants remained with their eyes closed throughout the session. The onset and offset of each block was cued by a brief (<1.1 sec) auditory instruction. Prior to each experiment, participants received detailed instructions regarding the various tasks at each mental condition and were told which auditory cue signified each condition. In the RS condition, each participant was instructed as follows: “silently say ‘one’ in a comfortable speed” (cue word: number one, cue duration: 1050 msec). In the VF condition, participants were instructed: “generate continuously and silently new words which start with the cue letter” (cue letters were either: a/b/g/r/n, cue duration: 500, 350, 650, 500, and 730 msec respectively). For the interspersed Rest periods, the participants were instructed: “rest as best as you can” (cue word: rest, cue duration: 620 msec). Presentation® software (Neurobehavioral Systems, Inc., Berkeley, CA) was used to deliver the auditory stimuli via the MR-compatible headphones.

Immediately following each of the two experiments, participants were interviewed in order to provide a brief description of the content of their experience. Particularly, they were asked after the RS experiment to describe freely their experience during the RS and the Rest conditions. After the VF experiment, they were asked, in addition to their experience during the Rest, about their experience during the VF condition, including rating of the level of success on a ten-point scale (1 = very low to 10 = very high, all participants reported scores above 6, mean 7.7 ± 2.3) to ensure compliance.

Finally, each participant performed with their eyes open a visual localizer experiment, which was a one-back four-category (faces, houses, objects, and patterns) visuomotor task (Goldberg et al. 2006). This localizer experiment was used to define the DMN regions for our group of participants (section Regions of interest analyses), as previously reported (Preminger et al. 2011).

### Respiration recording

A confounding effect, which might account for the observed reduction in BOLD signal during the RS condition compared to Rest, is a global BOLD signal reduction due to respiration differences between the conditions. In order to investigate this possibility, respiration was recorded simultaneously with the MRI scans. This was done for a subset of twelve participants, due to personnel limitations of the study (age 40.4 ± 7.8 years, four female), and was measured with real-time spirometry (AD Instruments), via nasal cannulas positioned in the participant's nostrils (Salter Labs). Analysis included five measures: respiration pace (breaths/min), inhale duration (sec), inhale peak (average inhale depth, measured in mV), inhale mean, and inhale integral (both in mV). Differences between conditions in each parameter were examined with paired *t*-tests, Bonferroni corrected.

### Imaging setup

Images were acquired on a 3 Tesla Trio Magnetom Siemens scanner, at the Weizmann Institute of Science, Rehovot, Israel. Functional T2*-weighted images were obtained with gradient echo planar imaging (EPI) sequence (TR = 3000 msec, TE = 30 msec, flip angle = 90°, FOV 240 mm, matrix size 80 × 80, scanned volume—46 axial slices tilted to the ACPC plane, of 3 × 3 × 3 mm voxels to cover the whole brain without gaps). Three-dimensional T1-weighted anatomical images were acquired to facilitate the incorporation of the functional data into the 3D Talairach space (1 × 1 × 1 mm resolution, 3D MP-RAGE sequence, TR = 2300 msec, TE = 2.98 msec, TI 900 = msec/9° flip angle).

### fMRI data analysis

fMRI Data were analyzed with the “Brain-voyager” software package (Brain Innovation, Maastricht, the Netherlands) and with complementary in-house software written in Matlab (Mathworks, Natick, MA). The functional images were incorporated into the 3D data sets through trilinear interpolation. Preprocessing of functional scans included 3D motion correction, filtering out of low frequencies up to two cycles per scan (slow drift), and spatial smoothing using a Gaussian kernel with a full width at half maximum of 6 mm.

Segments showing movement artifacts larger than 1 mm or sharp head movements were excluded from the analysis. The cortical surface in a Talairach coordinate system (Talairach and Tournoux 1988) was reconstructed for each participant from the 3D-spoiled gradient echo scan. The functional images were then superimposed on 2D anatomic images and incorporated into the 3D data sets through trilinear interpolation.

Statistical analysis/mapping was based on the general linear model GLM, with a regressor for each condition in the experiment. All regressors were modeled as box-car functions convolved with the hemodynamic response function. A hemodynamic lag of 3–6 sec was assumed for each participant. In order to account for nonneuronal contributions to the BOLD signal for each participant, six null predictors were added to the analysis, corresponding to head motion data in the relevant axes (three translational and three rotational vectors). The analysis was performed independently for the time-course of each individual voxel. Multi-subject analysis was based on a random-effect GLM. After computing the coefficients for all regressors (including the null regressors of the head motion), a Student's *t*-test between coefficients of different conditions (e.g., RS vs. Rest) was performed. Importantly, in order to minimize the possible differential effect of any immediate previous condition on the Rest, and to improve the signal to noise ratio, we used for each experiment the average of all the Rest periods, not only those who immediately preceded the VF or the RS conditions. Altogether, the RS experiment had 15 rest blocks, while the VF experiment had 20 rest blocks, which were used to calculate the mean-rest (“Rest”) for each of the respective experiments.

The multi-subject functional maps were projected on an inflated or unfolded Talairach normalized brain. Significance levels were calculated, taking into account the minimum cluster size and the probability threshold of a false detection of any given cluster. This was accomplished by a Monte Carlo simulation (cluster-level statistical threshold estimator in “Brain-voyager”), using the combination of individual voxel probability thresholding. The probability of a false-positive detection per image was determined from the frequency count of cluster sizes within the entire cortical surface. For the VF, a minimum cluster size of 98 voxels was significant. For the RS, a minimum cluster size of 116 voxels was significant. For presentation purposes, we used these values as cluster threshold for the corresponding maps.

### Regions of interest analyses

In all the regions of interest (ROIs) analyses described, ROIs of activation were defined for each subject individually, based on activation in an independent task. The R- IPL was defined from the visual localizer task, while the L-Broca was defined from the sentence-conjugation task. ROIs were identified as the activated voxels located within 10^3^ mm of the activity center (*P *<* *0.02, 890 ± 127 and 868 ± 187, anatomical voxels for R-IPL and L-Broca, respectively).

### Voxel distribution analysis

In order to establish a quantitative measure of the negative and positive activation values in the cortex for each task (RS and VF), we conducted a distribution analysis of all cortical voxels across all participants (37 177 ± 6086 functional voxels, 271 581 total anatomical voxels). After creating contrast maps for the relevant conditions using single subject GLMs (while accounting for head motions), we extracted the *t*-value of all the cortical voxels (using a cortical mask imported into Matlab). We then produced a histogram of *t*-values (negative and positive) for each participant, where each bar contained the number of voxels that acquired the value within the relevant bin (bins spanned from *t *=* *−6 to 6 in intervals of 1). The histograms of all the participants were averaged and the SEMs between participants were calculated, while taking into account the within-participant design (Morey 2008). In order to conduct a statistical test, we counted the negative and positive cortical *t*-values that exceeded the threshold (*P *<* *0.05) for each participant. We then calculated the percentage of negative and positive values for each participant in each of the two tasks (VF and RS), and performed paired *t*-tests between positive and negative percentages within each task. Finally, we created an ‘antagonism index’ for each participant [(positive − negative) / (positive + negative)] and conducted a paired *t*-test between the index values of the two tasks.

### Concatenation of functional data

In order to examine whether the difference between the VF and RS conditions may be due to different Rest periods averaged across the two experiments and taken as baseline in each of the statistical analyses, we concatenated the data of the two experiments into a single time course, and reanalyzed the data of the combined scans. To minimize DC shifts (biased mean values) in BOLD signals across scans, the concatenation was performed after subtracting the mean of the raw signal in each scan from each voxel in the brain. The single subject GLM was then performed on the unified data, while taking the Rest periods of both experiments as baseline condition. The head motion graphs were also concatenated and used as null predictors in the unified GLM.

### Behavioral test

The verbal reports collected from the participants at the end of the experiments were qualitative in nature, and intended just to make sure that the participants followed the instructions. However, after analyzing the fMRI data, we decided to obtain a more quantitative data of the experiences during the RS and Rest conditions, in order to quantitatively search for possible links between different types of behavioral responses (e.g. “Extrinsic” vs. “Intrinsic”). To this end, we conducted a separate behavioral experiment.

#### Participants

Thirty healthy participants took part in the additional behavioral experiment; all were research students (age 26 ± 9 years, 17 females). The behavioral test was approved by the Interdisciplinary center Herzliya Ethics Committee.

#### Experimental design

Participants were asked to sit quietly with their eyes closed, and engage in one of two conditions: either quiet rest (“Rest”), or repetitively saying “one” internally (“RS”). The length of the conditions was 1 min, largely equal to the sum of five fMRI blocks of 21 sec each. The order of conditions was counterbalanced between participants. Immediately after each condition, an inquiry form was administered. The inquiry form, prepared by A. B-O, consisted of 27 questions regarding different aspects of their internal experience. All questions started with “To what extent did you experience…” and continued with possible experiences, such as ‘thoughts’ or ‘emotions’. Each question received a rating ranging from 1 = low to 5 = high.

#### Inquiry form analysis

Out of the 27 questions, 11 questions were found to be ambiguous (i.e., unclear, understood differently by different participants, such as ‘To what extent did you experience success?’) and were excluded from further analyses. Four authors served as judges for categorizing the remaining questions. The chosen categories were: “Thoughts” (seven questions) and “Sensations” (three questions). These categories served for presentation of the results.

## Results

### The repetitive speech (RS) effect

**Figure 2:**
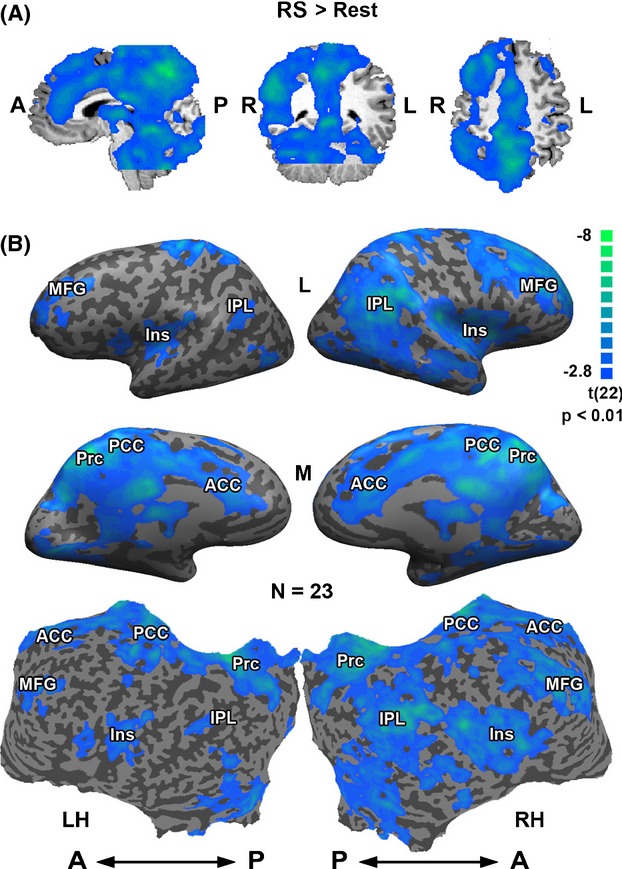
Decrease in fMRI BOLD activation during RS condition. Activation maps (RS vs. Rest; multi-subject random-effect GLM analysis, *P *<* *0.01), in: (A) 3D formats, Sagittal, coronal and transverse views (left to right); (B) inflated (top and middle) and unfolded (bottom) formats. ACC, anterior cingulate cortex; Ins, insula; IPL, inferior parietal lobule; MFG, middle frontal gyrus; PCC, posterior cingulate cortex; Prc, precuneus; LH, left hemisphere; RH, right hemisphere; A, anterior; P, posterior. Note the unidirectional negative responses without any significant positive BOLD. Blue–green regions represent areas that showed reduced activation.

**Figure 3:**
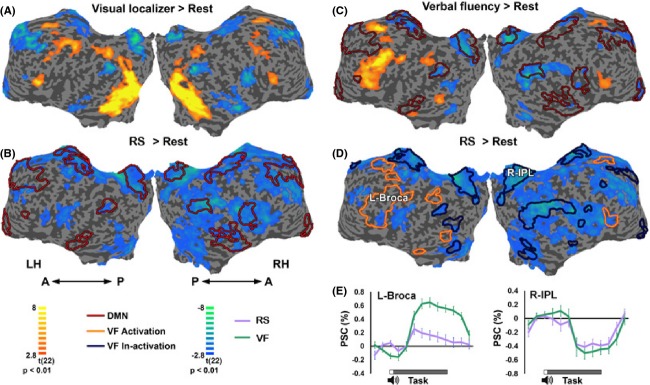
Comparison of Repetitive Speech (RS) and Verbal Fluency (VF). (A–D) Activation maps (multi-subject random-effect GLM analysis (Monte Carlo corrected, *P *<* *0.01)). Yellow–orange regions represent areas that were preferentially activated, while blue–green regions represent areas that showed reduced activation. (A) Visual localizer versus Rest (*n *=* *21); (B) RS versus Rest. Red contours – DMN regions taken from A (*n *=* *23); (C) VF versus Rest (*n *=* *23); (D) RS versus Rest with VF positive (orange) and negative (cyan) contours taken from C. LH, left hemisphere; RH, right hemisphere; A, anterior; P, posterior. (E) Averaged time courses (M ± SD) during the RS and VF conditions (*n *=* *23), sampled from right IPL and left Broca (in D). Negative-sustained activations (below baseline) in right IPL for both VF [*t = *−3.4, *P *=* *0.01, Bonferroni corrected] and RS [*t = *−3.9, *P *=* *0.002, Bonferroni corrected]. For the left Broca, VF showed a strong significant positive response [*t = *11.0, *P *=* *10^8^, Bonferroni corrected], while RS showed a marginally significant positive response [*t = *2.6, *P *=* *0.06, Bonferroni corrected]. Block period (21 sec, green bar) and auditory cue (red square) are marked below the graphs.

Comparing the RS with Rest revealed a significant reduction in BOLD signal during the RS across a number of cortical regions (Fig.2, Table 1984), including the bilateral posterior and anterior cingulate cortex and precuneus, as well as the right inferior parietal lobule, medial frontal gyrus, and insular cortex. Of particular importance is the observation that no sub-cortical (Fig.2A) or cortical (Fig.2B) areas exhibited a significant positive activation above Rest during the RS. Interestingly, the suppressed cortical regions showed substantial overlap with the DMN, independently defined in the same participants by a visual localizer task (Fig.3A and B).

**Table 1:** Regions that exhibited lower activity during RS relative to Rest. Coordinates are reported in Talairach space. The displayed *t*-values are associated with the area's lowest hemodynamic response during RS relative to Rest blocks

Anatomical location	BA	Coordinates			*t*-value
		X	Y	Z	
Frontal					
L. middle frontal gyrus	9	−32	33	35	3.46
R. middle frontal gyrus	8	40	23	42	4.97
R. superior frontal gyrus	10	24	49	24	4.97
Parietal					
R. inferior parietal lobule	40	54	−49	33	5.51
R. precuneus	7	1	−69	48	5.85
L. precuneus	7	−5	−69	48	5.85
Limbic					
R. posterior cingulate	31	14	−40	40	5.81
R. parahippocampal gyrus	30	−10	−43	2	3.42
B. cingulate gyrus	23	0	−25	28	4.98
B. anterior cingulate	32	0	30	23	4.44
Sub-lobar					
R. insula	13	33	10	7	4.44
R. insula	13	46	−28	23	5.51
L. insula	13	−34	−24	9	3.99
Temporal					
L. middle temporal gyrus	39	−48	−61	21	3.21
Occipital					
R. fusiform gyrus	37	25	−83	0	3.32

All coordinates emerged at a threshold of *P *<* *0.01, number of voxels = 116, Monte Carlo corrected. R, right; L, left; B, bilateral; BA, Brodmann area.

### Comparing repetitive speech (RS) to verbal fluency (VF)

To examine whether a unidirectional reduced activation profile was a common property of language generation tasks, we compared the map of the RS vs. Rest contrast with that of the VF vs. Rest contrast (Fig.3C and D). In contrast to the RS, VF produced an intense and significant positive activation mainly in frontal areas, including left Broca, dorsolateral prefrontal cortex, and inferior frontal gyrus (BA 45, 46 and 47, respectively), as well as a number of regions showing significant negative responses, largely including the DMN (Fig.3C). In contrast, the RS task showed in language-related regions a marginally significant activation (Fig.3D and E). Importantly, despite the massive difference in task-positive activation, the depth of the negative responses appeared to be relatively similar in both the VF and RS conditions (Fig.3D). We have also conducted a direct *t*-test between the VF and RS maps (shown in Fig.3C and D). The resulting map is shown in Figure S2. Also, note that the differences are strongest around the left lateralized language regions (i.e., Broca), indicating that both conditions generally exhibited a similar level of reduction in DMN regions, but only VF produced preferential activation during the VF task in language regions. This supports again the finding that RS shows a marked reduction in activity in all affected regions, including the language areas.

### Voxel distribution analysis

**Figure 4:**
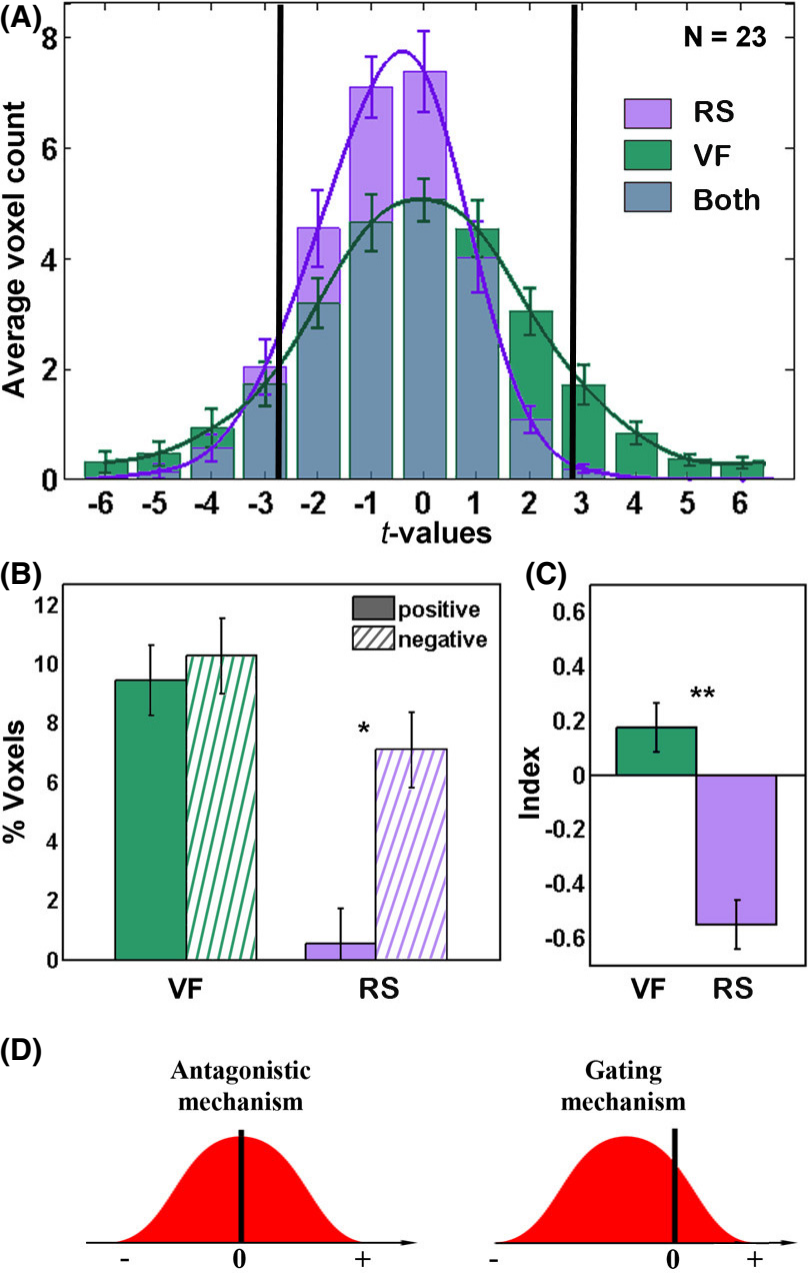
Distribution of t-values in cortical voxels during Repetitive Speech (RS) and Verbal Fluency (VF) tasks. (A) Mean (±SEM) cortical voxels distribution analysis (*n *=* *23, *t*-values for RS/VF vs. Rest). The vertical black lines denote the threshold used for visualization purposes in Figs3; (B) Percent of significant (*P *<* *0.05) positive and negative voxels in the RS vs. VF; (C) “antagonism index” (positive − negative) / (positive + negative). Error bars in (B) and (C) indicate confidence interval for means, while taking into account the within-participant design. *P <* *0.05, **P <* *0.005, Bonferroni-corrected post hoc *t*-test; (D) Hypothetical voxel activation profile predicted by the antagonistic and gating mechanisms.

To obtain a quantitative estimate of the relative levels of positive and negative activations during the RS and VF conditions throughout the cortex, we conducted a voxel distribution analysis of the entire set of cortical voxels across all participants. In this analysis, we compared the frequency distribution of the positive and negative BOLD activation (indexed by significance level) relative to Rest. The counting of the activated/deactivated voxels enables to present the inter-subject variability (seen as error bars), which cannot be seen in the multi-subject GLM maps. Importantly, there was no cluster-based threshold applied for the count. The Monte Carlo thresholds are only applied for visualization purposes on the maps themselves. However, it renders the direct comparison by the eye between the maps that are inaccurate. In contrast, the distribution analysis bypasses exactly this problem by providing the reader with threshold-independent data. To make this issue even clearer, we inserted into Fig.4A two lines showing where the threshold in the maps pass, so the reader can see the voxels that were actually shown in the maps. As can be seen (Fig.4A), the RS activation distribution was highly skewed toward negative values compared to the VF task. A simple count of significant positive and negative voxels (voxels with absolute *t*-value above 2.819, *P *<* *0.05) revealed that during RS, there were significantly more negative than positive voxels (*P *=* *0.014, paired *t*-test), as opposed to the similar voxel count during VF (*P *=* *0.8, paired *t*-test; Fig.4B). The relative imbalance of positive and negative voxels was calculated using an “antagonism index” [calculated by: (positive − negative) / (positive + negative)]. This analysis revealed a highly significant difference in antagonism between the RS and VF conditions (*P *<* *0.0005, paired *t*-test) (Fig.4C).

### Concatenation analysis

The second issue – this is an important consideration to notice. Indeed, we were concerned about the possibility of differences in rest baselines across the two conditions. The effect remained significant even after the concatenation. It could be argued that part of the difference between the activation patterns during the VF condition and the lack of activation during the RS condition may be due to differences in the average BOLD signal level during Rest periods taken as baseline in each experiment. Another concern is the possibility of task “leaking” into the rest. To examine the possible effect of such “leaking”, we conducted a control analysis where we concatenate the rest periods from both our experimental runs into a single time-course and reanalyzed the data of the combined scan, using this combined set as a baseline. That way if indeed there was “leaking” of the task activity into the rest, it should affect both our conditions equally. The new maps, comparing each condition to the concatenated rest, could be seen in supplementary Figure S1. The effect remained significant even after the concatenation: a significant difference between the antagonism index values for the two conditions was retained (*P *<* *0.05), indicating that the difference between the rest periods calculated as baseline for VF and RS could not explain all the difference, although it might account partly for some of this effect, as level of significance of the antagonism index was reduced.

### Respiration analysis

**Figure 5:**
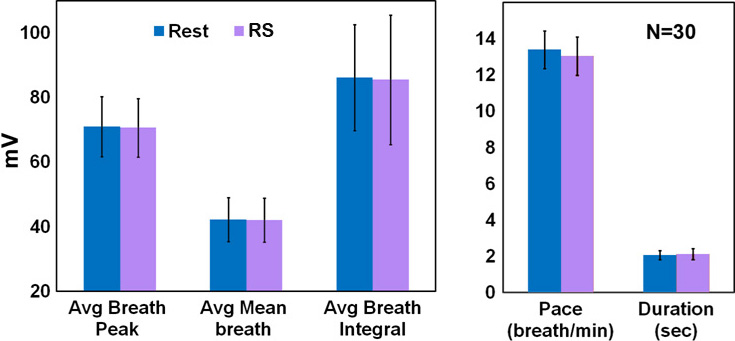
Nasal breathing analyses. The comparison of RS and Rest (*n *=* *12), in five different breath parameters (Mean ± SEM). There were no differences between the conditions in any breath parameter.

Another potential confound which may underlie the widespread negative BOLD signal during the RS condition could be respiration. Although change in breathing was previously shown in some meditation studies (e.g. Farrow and Hebert 1982; Badawi et al. 1984; Lazar et al. 2000), this is not always the case (Pagnoni 2012). To rule out this possibility, five breathing parameters were measured in a subset of 12 participants, using an airflow sensor during the MR scan. Importantly, no significant difference was observed in participants' breathing during the RS and Rest conditions (Fig.5).

### First person reports

The first person reports collected from the participants at the end of the RS experiments were diverse and qualitative in nature. Typical examples describing the experience during the Mantra condition were: “it was easy to do, but monotonous and boring”; “it was nice, somewhat focused, I visualized number one while repeating it verbally”; “it was similar to the rest, just deeper, there were no thoughts”; and “it was more relaxed and easy compared to the rest period”.

**Figure 6:**
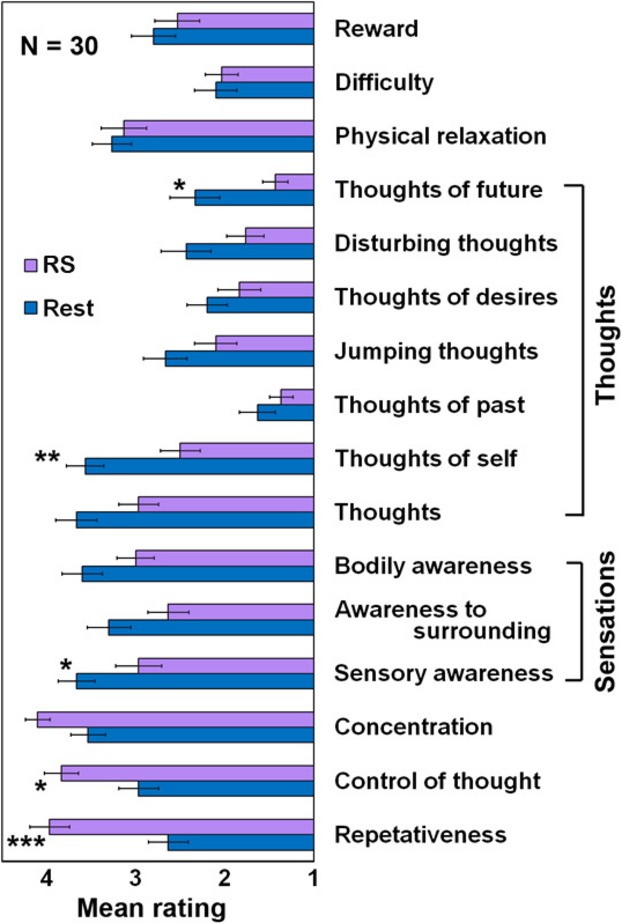
Full behavioral inquiry form scores. Scores of 17 questions, each starts with “to what extent did you experience…” (1 = low, 5 = high, Mean ± SEM, *n *=* *30). The categories ‘Thoughts’ and ‘Sensations’ are marked. *P <* *0.05, **P <* *0.01, *^**^P <* *0.001, Bonferroni corrected. Notice reduction in both ‘Thoughts’ and' Sensations' scores for the RS compared to the Rest.

**Figure 7:**
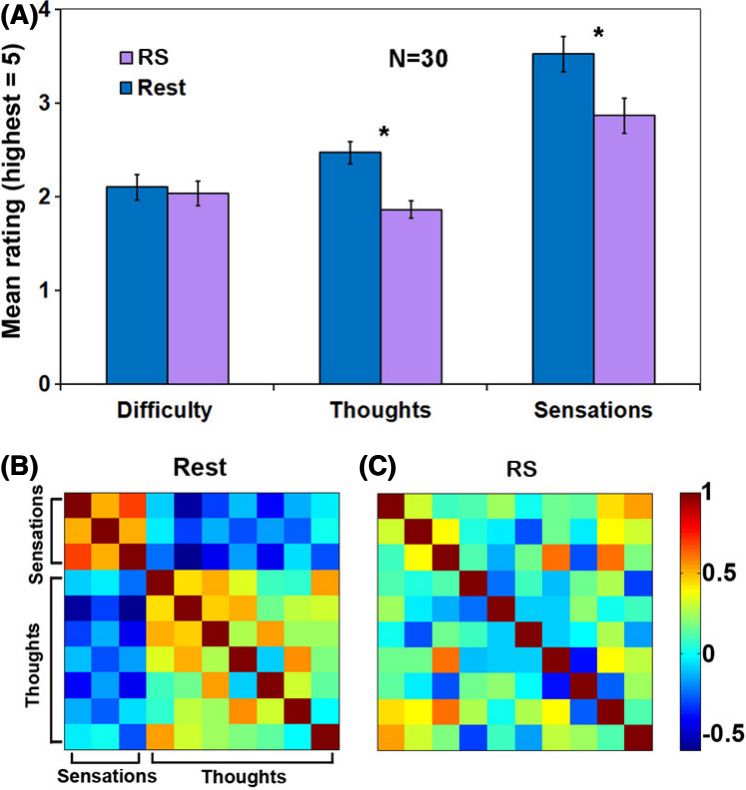
Behavioral questionnaire scores and correlations. (A) Mean ± SEM scores of three question categories (for details, see *15*): For Difficulty, Thoughts, and Sensations. *P <* *0.0005; (B–C) Pearson correlation matrices between ratings of Thoughts and Sensations for the Rest (B) and RS (C) conditions. Color scale denotes correlation values.

To obtain a more detailed comparison of the cognitive states of subjects during the RS and Rest conditions we conducted a follow-up behavioral experiment that included detailed interviews (see methods). The results of the separate behavioral test are summarized in Fig.6. For most parameters, such as thoughts of self or of future, the RS condition was reported to result in a reduction compared to the Rest condition, albeit only several parameters survived the Bonferroni-corrected significance threshold. However, for a few parameters, particularly control and concentration, the reverse was true. The chosen categories of experience for further analyses were: “Thoughts” and “Sensations” (Fig.6). These categories' mean served for further statistical analyses, presented in Fig.7. Notably, “Thoughts” were significantly (*P *<* *0.0001, paired *t*-test) less frequent during the RS condition. Similarly, “Sensations” were reported to be significantly (*P *<* *0.0005, paired *t*-test) less frequent during the RS condition. Examining the Pearson correlation matrices (Fig.7B and C) revealed an anti-correlation between “Thoughts” and “Sensations” during Rest (*r *=* *−0.24 ± 0.17). However, during the RS condition, this anti-correlation markedly reduced (*r *=* *−0.04 ± 0.27).

## Discussion

### Repetitive speech elicits widespread deactivation, centered mainly on the default mode network

Our fMRI results show that a surprising level of widespread negative responses can emerge in the human cortex when participants perform a repetition of a single word. This task recapitulates the motor aspects of commonly practiced Mantra practices but excludes the commonly practiced training context with its possible unique cognitive and emotional impacts.

The anatomical location of the negative responses during the RS condition substantially corresponded to the DMN, a network implicated in self-oriented thoughts (Gusnard et al. 2001; Raichle et al. 2001; Goldberg et al. 2006; Buckner et al. 2008; Preminger et al. 2011) and “mind wandering” (Smallwood and Schooler 2006; Mason et al. 2007; Christoff et al. 2009). Additionally, deactivation was seen in the insula, specialized for interoception (Craig 2002), as well as the anterior cingulate cortex. However, it is likely that different parts of the DMN may subserve different functions (e.g. Salomon et al. 2014). Thus, not surprisingly, the very specific RS task did not lead to a uniform inactivation of the DMN. For example, no inactivation was observed in the hippocampus formation – likely due to the minimal requirement for memory recall during this task.

It could be argued that the wide-spread reduction observed during repetitive speech may be a result of nonlocal effects, in particular changes in breathing patterns that could be induced by the monotonous task. However, our detailed analysis of such breathing patterns (see section Respiration analysis) failed to reveal any significant difference between the RS and Rest condition.

Finally, we found the same global reduction when comparing the RS to different Rest baselines – both in separate and concatenated scans – (see section Concatenation analysis) arguing against the possibility that the reduction was due to a comparison to a specific baseline condition.

### Proposing a ‘gating’ mechanism

Importantly, unlike most conventional task-related cortical activations (Raichle et al. 2001; Buckner et al. 2008) the broad negative responses during the RS condition occurred in the absence of corresponding significant positive activations. These results argue against a simple push–pull model of antagonistic relationship between cortical activation and inactivation as a source of the cortical inactivation during the RS condition.

Under a push–pull model, during attention-demanding tasks, task-specific cortical regions will be positively activated, while regions irrelevant to the task (e.g., the DMN) will be concurrently inhibited (Mckiernan et al. 2003; Fox et al. 2005; Golland et al. 2007; Chang and Glover 2009). The antagonistic model predicts a fairly balanced distribution of task-positive and task-negative activations (Fig.4D). Indeed such typical antagonistic profile was observed during the VF condition (Fig.4A).

However, during the RS condition, the voxel activation profile did not appear compatible with such a model – since it was highly skewed toward a deactivation profile, and failed to show a corresponding positive increase (Fig.4). What could be the source of such global task-negative responses during the RS condition? A plausible model ties these effects to a gating scenario. Gating can be conceptualized as a process by which minimal activation of a key “bottle-neck” region can inhibit wider competing processes (Marti et al. 2012). Under this hypothesis, even a slightly demanding language task such as RS, may trigger a nonlinear gating mechanism which deactivates all cortical networks that converge on the same bottleneck. An interesting analogous example can be found in the gate control theory, initially proposed by Melzack and Wall (1965) in the context of pain-processing (Dickenson 2002). In this case, a light touch on the skin is enough to reduce (or block) the flow of competing information flow (pain) through a common gatekeeper.

While the antagonistic model predicts a fairly balanced distribution of task-positive and task-negative activations, the gate model allows for instances of highly skewed distributions, that is, a condition in which the cortex is largely in a task-negative mode without a corresponding task-positive activation (Fig.4D). In support for such a proposition, the RS condition voxel activation profile is compatible with a gating mechanism (Fig.4).

From the experiments reported here, the identity of such a hypothetical gate-keeper could not be deduced. However, two plausible options for the locus of such a gate come to mind. One possible region might be a language area (e.g. Broca, BA 45), suggesting that the minor yet significant activation of language areas by the RS (Fig.3E) activates a nonlinear gating mechanism that ‘blocks’ the thought-related DMN, which might share a dependence on similar language networks. Another possible gate-keeper might be the dorsolateral prefrontal cortex (DLPFC, BA 46), which is adjacent to the Broca region. The DLPFC is part of the control system interposed between the DMN and sensory networks (Vincent et al. 2008), and it has been proposed that internal trains of thoughts are produced through cooperation between the DMN and the control network which helps sustain and buffer internal trains of thought against disruption by the external world (Smallwood et al. 2012). It could be that the DLPFC, engaged in the RS, may concurrently inactivate the DMN processing. However, further research is needed to elucidate the identity of the gate-keeper proposed here.

It is important to note that the antagonistic and the gating models are not mutually exclusive. One can envision that under certain task situations, networks will exhibit an antagonistic behavior, while in other circumstances they may exhibit a gating relationship. For example, it could be that networks that converge on a common gatekeeper will be turned off, while those that can operate in parallel will only exhibit an antagonistic behavior.

Finally, the behavioral results support and complement the BOLD fMRI results by pointing toward an experiential reduction in antagonism between “Thoughts” and “Sensations” categories of experience. Considering that the task-positive ‘extrinsic’ network includes the insula and the somato-motor area (Fox et al. 2005), regions widely accepted to be involved in proprioception and somatic processing (e.g. Craig 2002), sensations could be thought of as activating the extrinsic network. Thus, when examining the correlation matrices between “Thoughts” and “Sensations” during Rest (Fig.7B), the expected anti-correlation between “Thoughts” and “Sensations” is revealed. However, during the RS condition, there is a more global, nonantagonistic relationship between the task-positive and task-negative related experience during the RS condition.

Importantly, it should be cautioned that our inference about the cognitive manifestations of the fMRI results was limited by having the detailed behavioral reports obtained in a separate experiment.

### A comparison with commonly practiced Mantra meditation studies

Here, we isolated the effect of silent repetitive speech, which is used in most commonly practiced Mantra meditative practices, on brain activity. This intentionally omitted the wider context and spiritual orientations of commonly practiced Mantra practices. Hence, the comparison with commonly practiced Mantra studies should be done with caution. However, it seems that such a comparison is nevertheless of interest, given the prevalence of repetitive speech in common meditative practices.

It could be argued that the mantra condition blocks used here (21 sec) were too short to fully replicate the brain effects of the typically longer meditation periods used in real meditation practice. Indeed many meditative practices are characterized by lengthy periods of repetitive speech. However, it should also be noted that in previous studies of mantra meditation – the use of shorter periods was not exceptional (e.g. 30 sec in Engström and Söderfeldt 2010).

The only study which previously compared mantra meditation to the resting state was by Davanger et al. (2010). In this study, a continuous ACEM meditation (6 min) was compared to a 1 min resting interval. They reported increased activation in left superior temporal gyrus (BA 22) and left precentral gyrus (BA 6), regions used for language processing and motor control, respectively. Our study, in accord with Davanger et al. (2010), indeed revealed a slight activation over a language processing region (Fig.3E). However, this activation did not reach statistical significance, possibly due to the short block duration compared to Davanger et al. (2010). Importantly, Davanger et al. (2010) did not examine any decrease in activity during the meditation, which is the central finding of the present study.

Other previous studies (e.g. Lazar et al. 2000; Engström et al. 2010; Guleria et al. 2013) typically compared commonly practiced mantra meditation to various control tasks, supposed not to evoke an emotional response and yet be used in order to subtract language-related activations, such as silent pseudo-words and words repetition, or random generation of words (reviewed by Sperduti et al. 2012 and Tomasino et al. 2012). These studies generally demonstrated increased activity in various brain regions. A recent meta-analysis of mantra meditation studies concluded that activity clusters involve the right supramarginal gyrus, the somatomotor area bilaterally and the left postcentral gyrus, with no significant deactivations (Tomasino et al. 2012). These regions correspond to Wernicke's area, somatomotor and somatosensory areas, respectively. In contrast, here we explicitly mapped the effect of the RS aspect compared to the resting state, and report only significant deactivations. Our failure to reveal significant activations could be possibly explained by the shorter block duration in our study. With regard to deactivations, it is unclear why previous studies failed to report such effects. However, considering that the contrast in these previous studies was with language tasks – it is possible that the control tasks used in these previous studies actually produced a deactivation of DMN areas (as seen in our VF task, Fig.3C). Contrasting the meditation conditions with such tasks may mask the deactivations that may be induced by both conditions – in contrast to the RS vs. rest comparison used in the present work.

Finally, Xu et al. (2014) have reported enhanced activation in the DMN during nondirective mantra meditation (allowing mind wondering) contrasted with mantra concentrated meditation (that disrupts such mind wondering). However, during the directive – concentrated mantra meditation, no such significant enhancement in DMN activation was found.

### The ‘Mantra’ effect?

Here we report on the intriguing finding, obtained from BOLD fMRI, that repetitive speech is sufficient to induce a largely unidirectional and widespread cortical reduced activation. Such a skewed reduction in activation supports a hypothesized model of a global gating mechanism of cortical activity by the RS practice**.** Thus, it may provide a neurophysiological basis for the profound relaxing power of repetitive speech, as have been previously reported (e.g. Feuerstein 2003).

More generally, our fMRI results may reveal new insights into the relaxing power of Mantra recitation – a ubiquitous technique that has been practiced throughout human history, became widespread in the West in the 1960s, and is considered to be the most popular type of meditation worldwide today (Goleman 1988; Braboszcz et al. 2010). In accord with its Sanskrit translation as ‘an instrument of thought’, we show that one isolated component of this practice – the element of repetitive speech even in untrained subjects, causes wide spread reductions in cortical activity. These reductions overlapped large cortical regions, including the DMN. The DMN has been related to a variety of self-related processes, such as autobiographical recall, future planning, as well as ‘mind wandering’ and ‘stimulus independent thought’ (Smallwood and Schooler 2006; Mason et al. 2007; Buckner et al. 2008; Christoff et al. 2009). These functions have been previously linked to emotional stress (Smallwood et al. 2007, 2009; Killingsworth and Gilbert 2010; Fell 2012; albeit see Raichle 2006 and Bar 2009).

To sum, our study suggests a possible underlying neurophysiological explanation that may account, at least partially, for the relaxing power of Mantra recitation and show that repetitive speech has a readily observable fMRI signature.

## Conclusions

We describe here that repetitive speech, an easy cognitive task, is sufficient to induce a wide-spread unidirectional reduction in activation in the human cortex even outside the context and training of commonly practiced Mantra. This behavior appears to lead to a wide-spread activity reduction in brain processes which may compete with this task, including the thought-related default mode network. This surprising finding provides important new insights into a possible nonlinear gating mechanism of high level cognitive processes that could underlie the phenomena of purely task-negative cortical responses. By examining behavioral measures, we confirmed, in line with the cortical reduced activation, that repetitive speech induces a significant reduction in thought-related cognitive processes. Our study thus suggests a neuronal mechanism that may account for the uniquely calming effect of Mantra meditative practice, explaining its wide use across cultural and historical boundaries.

## Supporting Information

**Figure S1.** Comparison of Repetitive Speech (RS) and Verbal Fluency (VF) from the concatenation analysis.

**Figure S2.** T-test between Verbal Fluency (VF) and Repetitive Speech (RS).
