# Supplementary figures and images for: Repetitive speech elicits widespread deactivation in the human cortex: the “Mantra” effect?
Source: Brain Behav. 2015 May 4;5(7):e00346. doi: 10.1002/brb3.346 (PMC4511287; doi:10.1002/brb3.346)

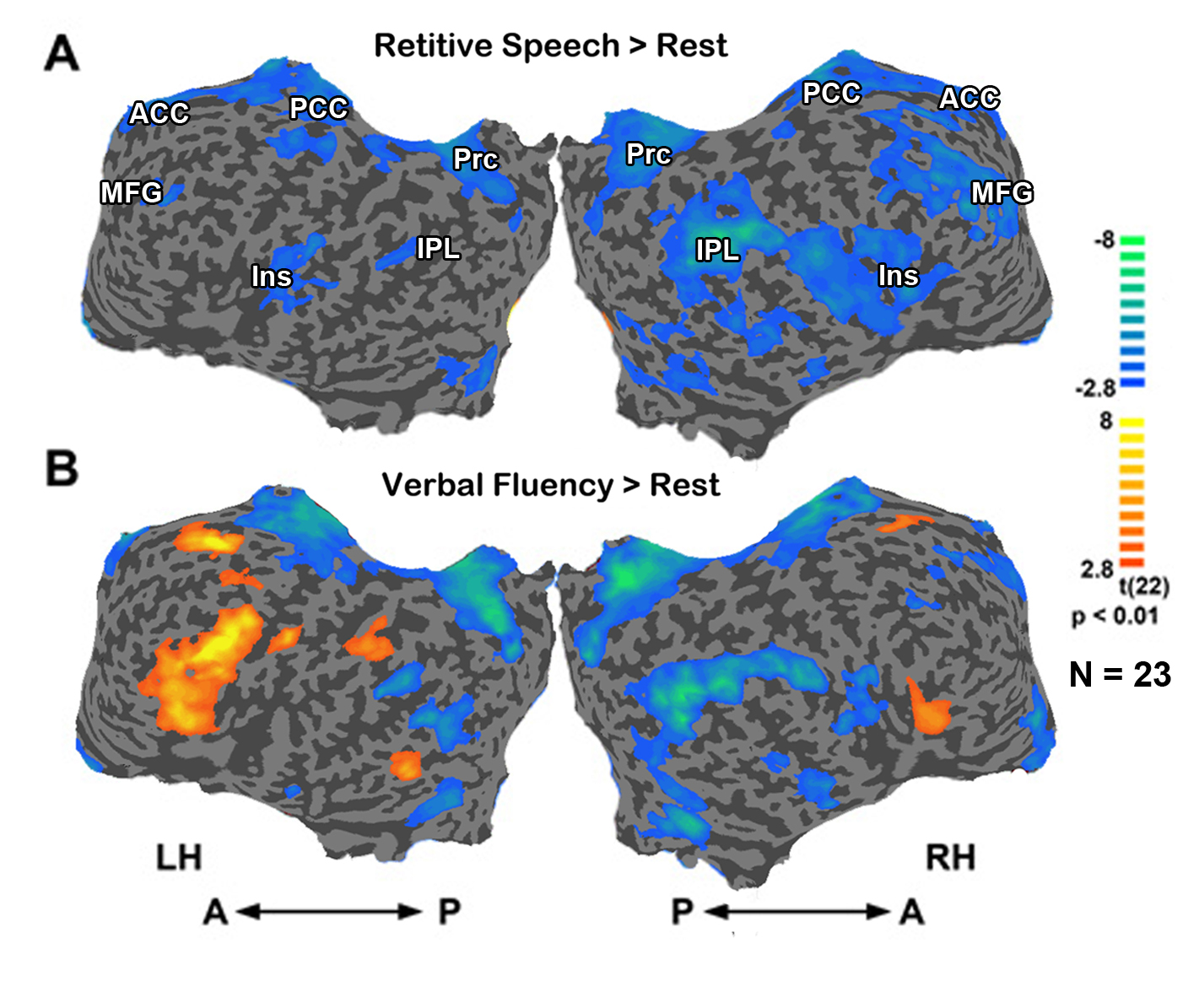

Supplement: Supplementary file 1 [file brb30005-e00346-sd1.tif]

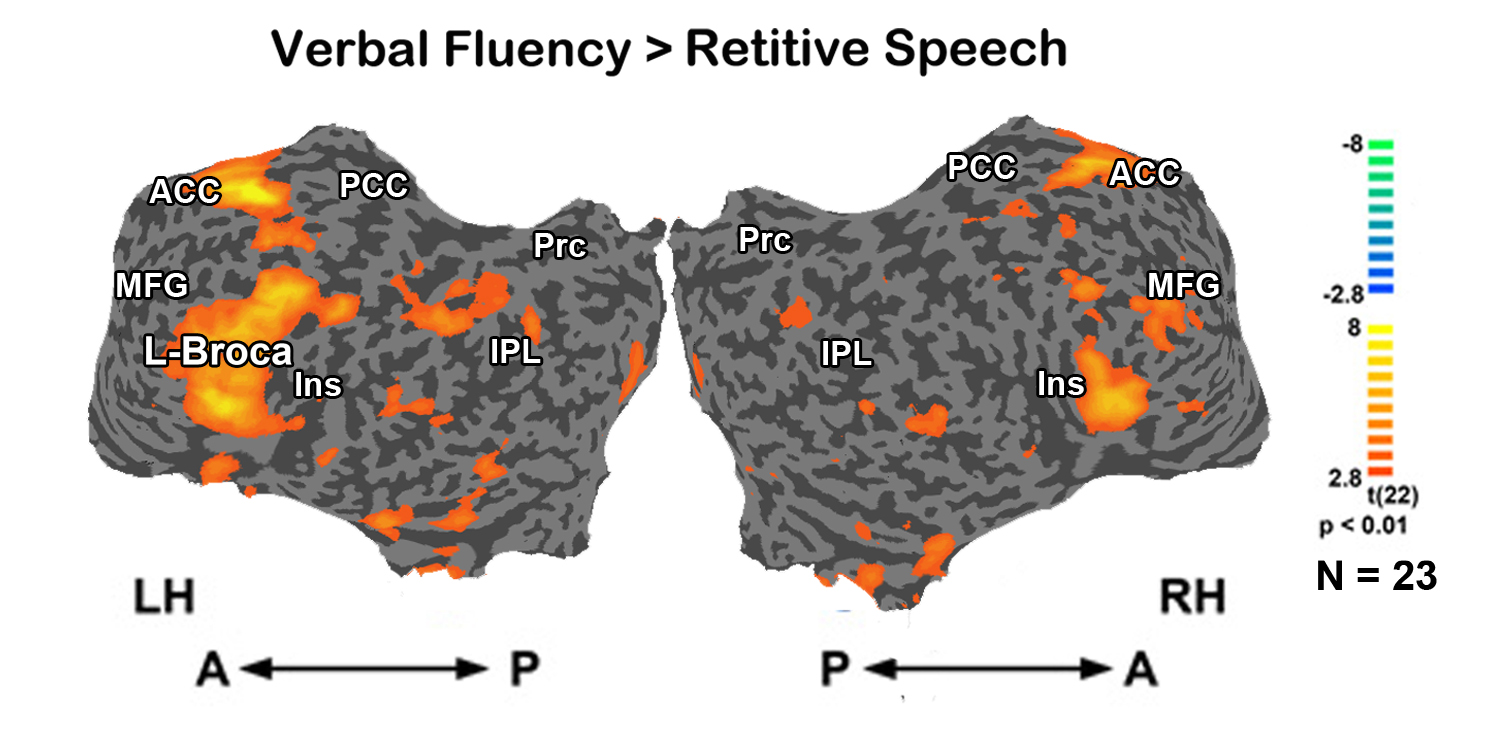

Supplement: Supplementary file 2 [file brb30005-e00346-sd2.tif]
